# Combining QCM and SERS on a Nanophotonic Chip: A Dual-Functional Sensor for Biomolecular Interaction Analysis and Protein Fingerprinting

**DOI:** 10.3390/nano15161230

**Published:** 2025-08-12

**Authors:** Cosimo Bartolini, Martina Tozzetti, Cristina Gellini, Marilena Ricci, Stefano Menichetti, Piero Procacci, Gabriella Caminati

**Affiliations:** 1 Department of Chemistry “Ugo Schiff”, University of Florence, Via della Lastruccia 3-13, 50019 Sesto Fiorentino, FI, Italy; cosimo.bartolini@unifi.it (C.B.); martina.tozzetti@unifi.it (M.T.); cristina.gellini@unifi.it (C.G.); marilena.ricci@unifi.it (M.R.); stefano.menichetti@unifi.it (S.M.); piero.procacci@unifi.it (P.P.); 2 Center for Colloid and Surface Science (CSGI), University of Florence, Via della Lastruccia 3-13, 50019 Sesto Fiorentino, FI, Italy

**Keywords:** FKBP12, electrodeposited nanomaterials, plasmonic silver nanoparticles, dual QCM-SERS sensor

## Abstract

We present a dual biosensing strategy integrating Quartz Crystal Microbalance (QCM) and Surface-Enhanced Raman Spectroscopy (SERS) for the quantitative and molecular-specific detection of FKBP12. Silver nanodendritic arrays were electrodeposited onto QCM sensors, optimized for SERS enhancement using Rhodamine 6G, and functionalized with a custom-designed receptor to selectively capture FKBP12. QCM measurements revealed a two-step Langmuir adsorption behavior, enabling sensitive mass quantification with a low limit of detection. Concurrently, in situ SERS analysis on the same sensor provided vibrational fingerprints of FKBP12, resolved through comparative studies of the free protein, surface-bound receptor, and surface-bound receptor–protein complex. Ethanol-induced denaturation confirmed protein-specific peaks, while shifts in receptor vibrational modes—linked to FKBP12 binding—demonstrated dynamic molecular interactions. A ratiometric parameter, derived from key peak intensities, served as a robust, concentration-dependent signature of complex formation. This platform bridges quantitative (QCM) and structural (SERS) biosensing, offering real-time mass tracking and conformational insights. The nanodendritic substrate’s dual functionality, combined with the receptor’s selectivity, advances label-free protein detection for applications in drug diagnostics, with potential adaptability to other target analytes.

## 1. Introduction

The accurate detection and quantification of proteins, such as FK506-binding protein 12 (FKBP12), are critical for biomedical diagnostics, drug development, and understanding molecular pathways in diseases like cancer and neurodegeneration. While conventional techniques like ELISA or mass spectrometry offer reliable FKBP12 protein analysis, they often lack the sensitivity, speed, or capacity for in situ molecular fingerprinting [1,2]. Surface-Enhanced Raman Spectroscopy (SERS) and Quartz Crystal Microbalance (QCM) have recently emerged as powerful complementary tools: SERS for providing unique vibrational fingerprints of biomolecules at ultra-low concentrations [3], and QCM for real-time, label-free mass quantification [4]. However, integrating these techniques into a single sensing platform remains challenging, requiring nanostructured surfaces that are simultaneously optimized for both SERS enhancement and QCM stability. Here, we propose a dual sensing strategy, combining QCM-based quantitative detection with SERS-driven molecular identification, designed specifically for the detection of FKBP12, a strategy that paves the way to a general protocol for label-free protein sensing.

FKBP12, formally known as FK506-binding protein 12, is a member of the immunophilin protein family, renowned for its diverse roles ranging from protein folding to cellular signaling modulation. Its complex involvement in physiological pathways and its implications in various diseases render FKBP12 a subject of profound scientific inquiry [2,5].

Although the intricacies of the molecular mechanisms underpinning FKBP12 diverse functionalities has not been fully unveiled, biomedical research has evidenced a pivotal role of FKBP12 in many pathologies such as autoimmune diseases [6,7], rejection after organ transplant [8,9], and neurodegenerations [10,11], as well as in cancer [12,13].

In this latter case, it has become increasingly clear that FKBP12 plays an important role in tumor genesis; for example, the expression of FKBP12 is the rate limiting factor that determines the responsiveness of a cell line or tissue to the anticancer drug rapamycin [13] and in general to FKBP12 inhibitors [14].

Recently, Zhang et al. [15] revealed that FKBP12 exhibits aberrant upregulation in most human cancers, with special emphasis in lung cancer. FKBP12 expression was found to be correlated with poor overall survival, disease-specific survival, disease-free interval, and progression-free interval in several cancers [16,17]. In particular, several works report that members of the FKBP protein family are selectively expressed in primary lung adenocarcinoma and [18] indicate FKBP12 as a valuable clinical biomarker for lung cancer [19]. Liu et al. also reported that that cancer cells with overexpressed FKBP12 are more sensitive to doxorubicin revealing how the level of FKBP12 may serve as a predictor of the responsiveness to anticancer treatment targeting the MDM2-p53 pathway [20].

Studies on the diagnostic values of FKBP12 in cancer treatment have been hindered by the scarce analytical methods proposed, since determining the precise concentration of FKBP12 in blood plasma or serum may be challenging due to its low abundance and the presence of other proteins at higher concentrations. Specific assays have been reported such as enzyme-linked immunosorbent assays (ELISAs) [21] or mass spectrometry-based measurements [22]; these methods, admittedly not recommended as diagnostic tools, are generally adopted for accurate quantification of proteins, but they are not commonly performed in routine clinical practice for the determination of FKBP12 due to the required specific capture antibodies and isotope-labelled internal standards for enhanced accuracy and precision [1,2].

The present work builds on a seminal study on a novel FKBP12 nanosensor, reported in a recent patent [23] that proposed a sensor platform that combined the specificity of a novel receptor for FKBP12 with the potential of nanofabrication to obtain a functionalization protocol easily coupled to a variety of analytical devices for the determination of FKBP12 in body fluids.

The nanostructured sensor unit for FKBP12 was fabricated using a compact monolayer of a novel FKBP12 receptor, GPS-SH1, synthesized after in silico design, to univocally target FKBP12 [23,24]. GPS-SH1 was covalently bound to the flat surface of a sensor chip of a QCM, and proper anchoring was obtained through the formation of a self-assembled monolayer (SAM), a nanofabrication that ensures precise control of surface density and thickness of the receptor molecule in the monolayer [25].

The nanosensor allowed for the quantification of FKBP12 in solution with a LOD of ca. 8 pM with linearity tunable from 10–50 pM to 100–800 pM. These figures are comparable to ELISA assays features [2] but without the immunoassay’s drawbacks. Good selectivity towards interferences such as BSA and IgG and long shelf-life were also obtained [24,26].

Here we present a novel nanosensor architecture that, stemming from our previous findings [23], exploits the advantages offered by the addition of a metal nanophotonic arrays with the twofold aim to increase the sensitivity of the method upon amplification of the surface area available for SAM formation and to combine quantitative QCM measurements with surface-enhanced Raman spectroscopy on the same sample for the identification of the protein fingerprint. While QCM enables label-free mass tracking, and SERS provides vibrational fingerprints of proteins, their integration faces challenges: designing nanostructured interfaces compatible with both techniques, preparing a surface-bound receptor selective for FKBP12 and decoupling overlapping signals from receptors and target proteins.

Here, we address these challenges fabricating robust arrays of silver dendritic nanostructures directly electrodeposited onto QCM sensors, enabling a unified platform for both techniques; the metal arrays were functionalized with a custom self-assembled monolayer (SAM) containing the GPS-SH1 receptor patented for its selective binding to FKBP12 [23]. When combined with silver nanostructures, QCM enables us to investigate nanoscale-level phenomena, ranging from biomolecular interactions to chemical sensing; silver nanostructures exhibit exceptional properties that make them ideal candidates for enhancing the capabilities of QCM-based studies [27]. Their large surface-to-volume ratio, tunable optical properties, and surface density make them attractive for sensing applications. By immobilizing Ag nanostructures onto QCM sensor surfaces, it is possible to probe a diverse array of surface interactions in real time and with high sensitivity. Furthermore, functionalizing silver nanostructures with specific ligands, ultrasensitive biosensors capable of detecting target analytes, such as proteins at extremely low concentrations, can be easily designed and fabricated. The binding events between the target analytes and the functionalized silver nanostructures induce changes in the mass and/or viscoelastic properties of the sensor surface, thereby enabling real-time monitoring and quantification of biomolecular interactions with high sensitivity.

On the other hand, SERS is a robust and versatile label-free technique used to detect and analyze molecules at very low concentrations. It combines Raman spectroscopy with the enhancement of signals from molecules adsorbed on nanostructured metallic surfaces, typically gold or silver [28].

The SERS effect relies on the increase in the electromagnetic field at the metal surface of a plasmonic nanostructure, which dramatically enhances the Raman signal of molecules placed in its close proximity [29,30]. The shape and size of the plasmonic nanoparticles as well as their bidimensional packing affect their optical properties, and large SERS effects are strictly dependent on the gap distance of adjacent nanostructures, termed “hot-spots”. SERS sensors based on noble metal nanoparticle arrays have gained increasing attention for their ultrasensitive detection of a wide range of analytes, from small molecules to large proteins, via surface-enhanced Raman spectroscopy (SERS). These arrays also provide additional sensing capabilities through localized surface plasmon resonance (LSPR), which is highly sensitive to subtle changes in the refractive index of the surrounding molecular environment [28].

Many different approaches have been proposed for the production of hot-spots on metal surfaces, including pioneering random aggregation of silver or gold nanoparticles induced by a salt [31] and more recent approaches that exploit external magnetic field to dynamically control the interparticle spacing of the nanoparticles in a monolayer at the hexane/water interface [32]. Previous studies revealed that compact 2D arrays of Ag nanocubes provided significant enhancement of the SERS signal [33,34], allowing for efficient SERS detection of proteins physisorbed on the AgNC-coated surface [35]; however, the fabrication of hot-spots with controllable density still remains a remarkable challenge. Furthermore, the ability to gather molecules in these regions in a homogeneous and reproducible manner is a major barricade for further diffusion of SERS as a routine analytical tool.

In the present work, we explored the direct electrodeposition of silver nanostructures on gold- and silver-coated QCM sensors. Proper tuning of the experimental conditions allowed us to obtain two different types of stable surface arrays of Ag nanoparticles (AgNP), i.e., nanoflowers and nanodendritic structures. The surface density, size and morphology of the electrodeposited AgNP were obtained by a variety of complementary techniques spanning from contact angle, reflectance spectroscopy and Scanning Electron Microscopy (SEM). Through systematic characterization of the two different Ag nanostructures using Rhodamine 6G as a SERS probe, we identified the Ag nanodendritic morphology as optimal for maximal Raman enhancement. The SAMs of silver nanodendritic arrays were functionalized in the QCM chamber with a SAM containing a 1:6 mixture of the receptor (GPS-SH1) and 1-Dodecanethiol (C_12_-SH) as an optimal molecular spacer that anchors the Ag surface while exposing the previously identified selective receptor group for FKBP12.

QCM measurements as a function of FKBP12 concentration in solution revealed a two-step Langmuir adsorption behavior for FKBP12, enabling sensitive mass quantification with a low limit of detection. Concurrently, in situ SERS analysis on the same sensor provided the vibrational fingerprints of FKBP12, resolved through comparative studies of the free protein, bulk receptor, and receptor–protein complex. Ethanol-induced denaturation confirmed protein-specific peaks, while shifts in receptor vibrational modes—linked to FKBP12 binding—demonstrated dynamic molecular interactions. A ratiometric parameter, derived from key peak intensities, served as a robust, concentration-dependent signature of FKBP12-receptor complex formation. This platform bridges quantitative (QCM) and structural (SERS) biosensing, offering real-time mass tracking and conformational insights for a known FKBP12 concentration in solution. Our approach bridges the gap between quantitative and structural analysis, offering a versatile tool for studying protein–ligand interactions in complex biological environments.

## 2. Materials and Methods

FK506 Binding Protein (FKBP12) expressed in *E. coli* (MW 11900) was supplied by CIRMMP (Florence, Italy). The purity of FKBP12 was greater than 95% determined by 1 SDS electrophoresis. The stock concentration (C= 2.4009 × 10^−5^ M) of FKBP12 in PBS (KH_2_PO_4_/K_2_HPO_4_ 0.5 M e NaCl 0.15 M, pH = 7.4) buffer was determined by UV absorbance at 280 nm (e_280_ = 9970 M^−1^).

GPS-SH1 was synthesized as previously described [23,24]. 1-Dodecanethiol (C_12_-SH), Thiol-polyethylene glycol (PEG-SH), AgNO_3_, and KNO_3_ were bought from Merck (Darmstadt, Germania) and used without other purifications. 

The solutions were prepared using pure water obtained by means of a reverse osmotic system (Milli-RO, Millipore GmgH, Darmstad, Germany) and by ion exchange and filtration (Milli-Q, Millipore Gmbh); the water resistivity is R= 18 MΩcm and the pH = 5.6. 

QCM sensors were bought from Q-sense (Biolin Scientific, Helsinki, Finland). The QCM sensors were treated with UV-Ozone cleaner for 10 min, and then they were submerged in piranha basic solution at 70 °C (H_2_O_2_ 30% (*w*/*w*) in H_2_O mixed with NH_4_Cl at a 1:3 ratio), washed with water, dried with N_2_, and treated again with UV-Ozone Cleaner for 10 min.

**Electrodeposition of silver nanostructures.** Electrodeposition was conducted at 25 °C using the Autolab PGSTAT30 working station (Methohm AG, Herisau, Switzerland). The electrolyte solution, consisting of AgNO_3_ 5 mM and KNO_3_ 1 mM, was introduced in an electrolytic cell with three electrodes configuration. An Ag/AgCl reference and platinum counter electrodes were used, while gold QCM support was used as a working electrode.

**Scanning Electron Microscopy.** SEM micrographs of QCM supports were obtained with Variable Pressure Hitachi SU3800 SEM equipped with Ultim Max 40 Analytical Silicon Drift EDS Detector (energy resolution of 127 eV at the Mn–Kα line), X4 Pulse Processor, and AZtecLive 6.0 software (Oxford Instruments NanoAnalysis, Abingdon, UK) with accelerating voltage of 15 kV. SEM micrographs of the ITO-coated glass slide were acquired using a field-emission scanning electron microscope (FEG-SEM, SIGMA, Carl Zeiss, Aalen, Germany) operated at an accelerating voltage of 5 kV and a working distance of approximately 7 mm, equipped with a secondary electron detector.

Images analysis was performed using the public domain NIH Image program (developed at the U.S. National Institutes of Health and available on the Internet).

**Contact angle measurements.** Contact angle measurements were obtained both by fast screening using a simple in-house setup that consists of a stand on which we place the samples and a holder to keep the camera aligned parallel to the sample surface and using an Automated Contact Angle Goniometer (Ramé-Hart, Inc., Mountain Lake, NJ, USA), including a fiber optic light source to reduce water evaporation effects a CCD camera for image capture and a computer controlled dispenser (Auto Pipetting System, Ramé-Hart) for drops of controlled volume (2 μL). Both right and left contact angles were obtained by image analysis every 5 s until spreading equilibrium was reached. Both methods provided similar results.

**UV-Vis spectrophotometer.** UV-Vis spectra of FKBP12 in solution were obtained using Nanodrop ONEc Microvolume UV-Vis spectrophotometer (Thermo Scientific™, Wilmington, DE, USA), volume 1–2 μL, and optical path = 1 cm. The concentration of the stock FKBP12 solution was calculated from the absorption at 280 nm (ε_280_ = 9970 M^−1^)

**Reflection spectroscopy.** The reflectance spectra were obtained using a spectrophotometer equipped with an integrating sphere (Lambda 35, Perkin Elmer, Milano, Italy). The wavelength range was 380–780 nm with a scanning speed of 240 nm/min. The calibration was performed using a perfect Lambertian diffuser. Spectral data were treated with the Kubelka–Munk equation [36] providing the absorption coefficient (α) from reflection data.

**Quartz Crystal Microbalance (QCM).** QCM measurements were performed with a QCM-Z500 (KSV Instruments Ltd., Helsinki, Finland) with impedance monitoring equipped with a thermoelectric (TE) module (Oven Industries, Mechanicsburg, PA, USA). The QCM sensors used in our experiments were provided by Biolin Scientific (Biolin, Espoo, Finland) and have a nominal resonance frequency of 4.95 MHz ± 50 Hz, with an AT-cut quartz wafer of 14 mm diameter and 0.3 mm thickness. The active sensing area is 0.785 cm^2^, and the electrode layer thickness is 100 nm for both gold- and silver-coated QCM sensors. The surface roughness of the coated electrodes is <1 nm for gold-coated sensors and <2 nm for silver-coated sensors, as provided by the manufacturer. The resonant frequency shift (ΔF) was simultaneously measured at the fundamental resonant frequency (F_0_) and at five odd overtones (n = 3, 5, 7, 9, 11), corresponding to resonance frequencies of F_n_≈5, 15, 25, 35, 55 MHz. The active area of the sensor was 0.785 cm^2^. The measuring cell was kept at T = 20.0 ± 0.1 °C with a Peltier element (Oven Industries Inc., Mechanicsburg, PA, USA) connected to the TE module; room temperature was 22.0 ± 0.1 °C. The QCM-Z500 response is sensitive to the mass and to the viscoelastic properties of the surface-bound layer. A decrease in ΔF_n_ coupled to low values of ΔD and invariance of ΔF_n/n_ indicate a rigid film, and in this case the measured frequency shift ΔF is linearly proportional to the mass density, Δm/A, of the deposited film according to the Sauerbrey Equation:

(1) $$∆F_{n}/n=-\frac{{2F}_{0}^{2}}{A\sqrt{\rho_{q}\mu_{q}}}∆m$$

where *n* is the overtone number and $\rho_{q}$ (2.648 g/cm^3^) and $\mu_{q}$ (1011 g/cm·s^2^) are the density and the shear modulus of the quartz crystal, respectively [37]. The data were also processed with the QCMBrowse 2.0 software (KSV Instruments Ltd., Helsinki, Finland) which, applying a Voigt-based model used for similar systems [35], directly provides the adsorbed mass, the thickness of the adsorbed layer, and the viscoelastic parameters of the system. 

**SERS and FT Raman Measurements.** SERS spectra are registered using a micro-Raman Renishaw RM2000 (Renishaw plc, Wotton-under-Edge, Gloucestershire, UK) equipped with an argon laser (emission λ = 514.5 nm) and diode laser (emission 785 nm). The laser beam is directed through an optical system and focused on the sample using microscope objective Leica DLML (5×, 20×, 50×, 100×). The scattered light returns, through a reverse path, into the spectrometer and passes through a notch filter, which does not transmit the wavelength of the laser radiation and allows the other spectral components to pass. The monochromator is a diffraction grating spectrograph (1200 lines/mm) that allows to obtain a spectral resolution of 4 cm^−1^. The filtered and spatially dispersed radiation is sent to a multichannel detector CCD RenCam (thermoelectrically cooled to −70 °C). The signals are analyzed with WiRE 5.0 software (Renishaw plc, Wotton-under-Edge, Gloucestershire, UK). FT Raman spectra were registered with a Multiram interferometer (Bruker, Billerica, MA, USA) equipped with 1064 nm excitation wavelength.

## 3. Results and Discussion

We propose a sensor platform that jointly benefits from nanoscale control of the receptor position and orientation and of the unique signal amplification properties offered by silver nanoplasmonic structures. This approach aims to enhance the sensitivity of micro gravimetric measurements and pave the way for the application of SERS in FKBP12 detection.

The integration of QCM and SERS techniques requires the design of a metal nanostructure array that is sufficiently dense to generate efficient hot spots for enhanced SERS detection. Simultaneously, the nanoparticle array must be stable enough to withstand solution flow within the QCM measurement chamber. Sensors based on noble metal nanoparticle arrays have gained increasing attention for their ultrasensitive detection of a wide range of analytes, from small molecules to large proteins, via surface-enhanced Raman spectroscopy (SERS) [34,35,38,39,40].

**Figure 1:**
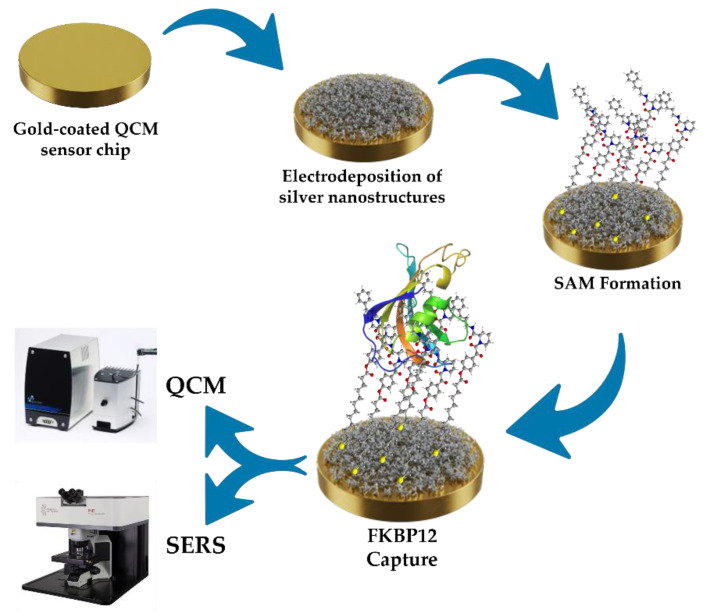
Schematic representation of the dual QCM-SERS detection platform proposed for FKBP12.

A schematic description of the proposed array and the detection approach proposed is reported in Figure 1.

As shown in Figure 1, the sensor consists of a QCM support further modified with silver nanostructures. These nanostructures are further functionalized with a self-assembled monolayer (SAM) that incorporates the receptor molecules specifically designed and synthesized to recognize and selectively bind the FKBP12 protein in solution.

### 3.1. Fabrication of SERS-Active Anisotropic Arrays of Silver Nanostructures on the QCM Sensor

We chose electrodeposition to obtain silver nanoarchitecture directly attached to the surface of the QCM sensor. Preliminary electrodeposition tests were performed on indium tin oxide (ITO)-coated glass substrates to avoid damaging the delicate and costly QCM crystals during parameter screening. A range of experimental conditions was explored by changing key parameters such as deposition potential, deposition time, and the composition of the electrolyte solution.

#### 3.1.1. Electrodeposition on ITO Surfaces

The electrodeposition procedure was developed from previous studies on conventional surfaces [41,42], employing a three-electrode configuration consisting of a working electrode (ITO-coated glass), a counter electrode, and a reference electrode (Ag/AgCl). The electrolyte solution consisted of an aqueous solution of AgNO_3_, which provides Ag^+^ ions that are reduced to metallic silver on the substrate surface during electrodeposition. Additional components such as KNO_3_ and PEG400 can be added to maintain constant ionic strength and, consequently, the conductivity of the solution and as a capping agent to control the growth morphology of the nanoparticles, respectively.

The influence of the main deposition parameters was clarified with respect to their impact on nanoparticle density, morphology, and growth control; details of the experiments are reported in the Supporting Information (Figures S1 and S2 in Supplementary Materials). We observed that an increase in AgNO_3_ concentration results in a higher density of electrodeposited nanoparticles. The shape and geometry of the nanoparticles is unaffected by AgNO_3_ concentrations, indicating that AgNO_3_ primarily modulates nanoparticle density rather than structural characteristics. Changing the deposition time influenced both the number of nanoparticles and their morphological evolution. Specifically, shorter or longer times resulted in less controlled growth, as expected; this suggests a time-dependent balance between nucleation and growth mechanisms. Modulation of the deposition potential enabled the formation of distinct nanostructures, highlighting its critical role in driving different growth pathways. Additionally, the presence of PEG400 as a capping agent was essential to direct the crystallographic growth of the nanoparticles. PEG400 enabled precise morphological control, yielding highly symmetrical structures such as nanoprisms and nanorods. In its absence, the growth process resulted in less regular and more aggregated morphologies.

By varying the deposition parameters, two distinct silver nanostructures were obtained on the surface of ITO-coated glass: silver nanoflowers (AgNFs) and silver nanodendrites (AgNDs). AgNFs were composed of nanoparticle aggregates with a radial arrangement resembling floral patterns and were obtained using PEG_400_ as capping agent. A key feature of these nanostructures is the extremely short interparticle distance (approximately 5–10 nm), which generates a high density of plasmonic hot-spots, making them highly promising candidates for SERS applications (Figure S1 in Supporting Materials). AgNDs were obtained under low constant potential using AgNO_3_/KNO_3_ as electrolyte solution; these structures exhibit branched morphologies and high surface areas with a high density of hot-spots. SEM images (Figure S2 in Supplementary Materials) show that longer deposition times allow the formation of well-developed dendrites, while shorter times resulted only in anisotropic nanoparticles. This behavior supports a Diffusion-Limited Aggregation (DLA) growth mechanism [43]. In the DLA model, the formation of dendritic structures takes place through nucleation followed by directional growth, mainly along the (111) crystal plane. At first, the particles grow into nanorods along (111), which then evolve into nanowires which then develop secondary and tertiary branches. The resulting dendritic architecture is governed by both ion diffusion and local aggregation, although the diffusion of silver ions in solution is the kinetic limiting factor [43].

#### 3.1.2. Electrodeposition on QCM Sensors

The procedure selected from the experiments on ITO slides was applied to QCM supports. Electrodeposition was carried out on three types of QCM supports, ITO coated (ITO QCM), gold coated (Au QCM), and silver coated (Ag QCM), all with a nominal resonance frequency of 5 MHz.

For the electrodeposition on QCM supports, two different protocols were employed with specific morphological targets: the first aimed to reproduce the silver dendrites (AgNDs) previously obtained on ITO-coated glass, while the second was designed to generate nanostructures similar to silver nanoflowers (AgNFs). In the first procedure, a low constant potential of –0.06 V was applied for 10 min in an aqueous solution containing 5 mM AgNO_3_ and 1 mM KNO_3_. In the second procedure, a constant potential of –0.9 V was applied for 100 s using a 0.5 mM aqueous solution of AgNO_3_ adding 5 mg/mL of PEG_400_. After deposition, all QCM supports were thoroughly rinsed with Milli-Q water and dried under a nitrogen stream.

**Figure 2:**
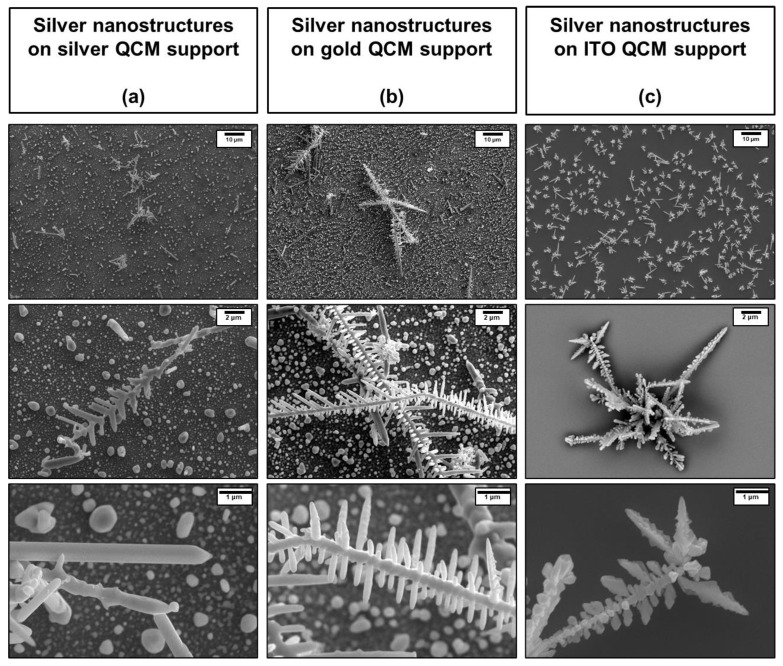
SEM images of QCM crystals electrodeposited using the first electrodeposition procedure described in the text: (**a**) silver QCM support with silver nanostructures, (**b**) gold QCM support with silver nanostructures, and (**c**) ITO QCM support with silver nanostructures.

Figure 2 shows Scanning Electron Microscopy (SEM) images, acquired at different magnifications, of the QCM substrates on which silver nanostructures were electrodeposited using the first electrodeposition procedure. As observed in the SEM images, this protocol enables the formation of dendritic silver nanostructures on silver-, gold-, and ITO-coated QCM substrates. The SEM analysis reveals the growth of silver nanodendrites (AgNDs), with their morphology and structural complexity, changes according to the underlying substrate. Whereas on ITO (Figure 2c), the surface is uniformly covered with silver dendrites, on Ag QCM (Figure 2a) and Au QCM (Figure 2b), dendritic growth occurs only in localized regions, coexisting with anisotropic silver nanoparticles (AgNPs) and areas free of any nanostructures. Furthermore, we observe that the dendritic structures formed on Au QCM are more extensive and structurally complex than those on Ag QCM. The dendritic nanostructures grown on gold QCM support exhibit extended lengths exceeding 5 µm, with tip cross-sections as small as approximately 30 nm. In addition to the dendritic formations, SEM images also reveal the presence of quasi-spherical AgNPs with diameters ranging from approximately 150 nm to over 900 nm (Figure S3). Based on the top-view SEM images, we estimate that the thickness of the deposited silver layer is below 1 μm in regions with dendritic structures, while in areas covered by quasi-spherical nanoparticles, it can be assumed to roughly correspond to their diameter, ranging from 150 to 900 nm. To further quantify the surface distribution of dendritic nanostructures and nanoparticles, SEM images of Au QCM, with dimensions of 126 × 94.5 μm (corresponding to an area of approximately 11.9 × 10^3^ μm^2^) were analyzed with NIH Image software (see Materials and Methods). The analysis indicates that approximately 13.9% of the surface is covered by AgNDs and 26.2% by AgNPs, while the remaining 59.9% corresponds to exposed gold surface without nanostructure coverage.

In contrast, when the second electrodeposition procedure was applied to silver- and gold-coated QCM substrates, only a small number of silver nanoparticles (AgNPs) were formed, randomly and unevenly distributed across the surface (Figure S4 in Supplementary Materials), no well-defined nanostructures were observed, and the characteristic flower-like morphology obtained on ITO-coated glass substrates (Figure S1 in Supplementary Materials) could not be reproduced. These results indicate that the second deposition protocol is not effective for forming AgNFs on metallic QCM surfaces.

In conclusion, stable and reproducible arrays of silver nanostructures were successfully obtained on QCM sensors, resulting in surfaces partially covered by silver nanodendrites and anisotropic silver nanoparticles. Populations of dendrites and nanospheres coexist on both Au- and Ag-coated QCM substrates; however, dendritic structures are more abundant, extended, and morphologically complex on the gold QCM supports compared to the silver ones. For simplicity, the gold QCM substrate containing both silver nanodendrites and nanoparticles has been referred to as AgNDs@Au despite the presence of both nanostructure types on the surface.

**Figure 3:**
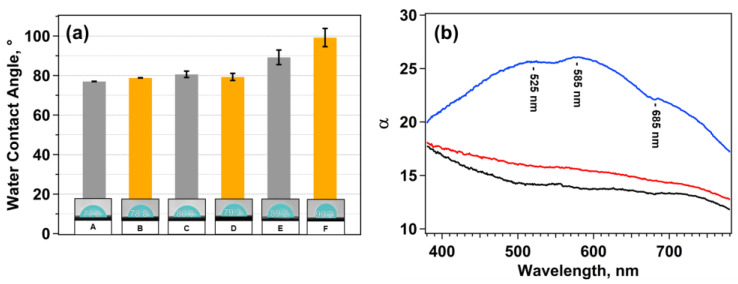
(**a**) Water contact angle measured on bare silver and gold QCM substrates (columns A and B), deposited using either the first (columns E and F) or the second electrodeposition procedure (columns C and D). Three water droplets (2 µL) were dispensed on each substrate, and the mean contact angle was calculated by averaging the left and right sides. The reported error corresponds to the standard deviation. (**b**) Absorption coefficient (α) of silver nanostructures on gold QCM support deposited with the first procedure (blue line), of gold QCM support deposited with the second procedure (red line), and of gold bare QCM support (black line).

Figure 3a reports the water contact angles (WCAs) measured on gold and silver QCM substrates deposited using the first or the second electrodeposition procedure. Wettability was investigated by dispensing three 2 µL water droplets at different locations on each QCM substrate. The reported WCA values for each substrate represent the average of the left and right contact angles measured for each droplet, and the associated error is given as the standard deviation. To evaluate the wettability changes induced by electrodeposition, the contact angles were also measured on bare gold and silver QCM substrates.

The results show that the second electrodeposition procedure induces only minor variations in the contact angle. This observation is consistent with the SEM images (Figure S3), which reveal an irregular deposition of few silver nanoparticles on both silver- and gold-coated QCM substrates. Consequently, the wettability is essentially unchanged compared to the bare QCM supports. In contrast, the gold and silver QCM supports prepared with the first procedure exhibit a significant increase in the contact angle. In particular, AgNDs@Au, characterized by the coexistence of silver nanoparticles (AgNPs) and silver nanodendrites (AgNDs), reaches a WCA close to 100°, while the silver substrate shows a WCA of approximately 90°. These results also align with the SEM observations: dendritic structures grown on gold QCM support are more extended and branched than those on silver QCM support, resulting in higher surface hydrophobicity. This behavior can be explained by the Cassie–Baxter model [44], according to which complex nanostructures such as dendrites trap air pockets between the water droplet and the surface, creating a heterogeneous wetting regime and enhancing the contact angle. However, the measured WCAs remain lower than those typically observed for superhydrophobic dendritic surfaces [45,46] due to the simultaneous presence of silver nanoparticles, which reduce the superhydrophobic effect.

The uniformity of the surface, and thus of the deposition, can be assessed by analyzing the standard deviation of the measured WCAs. Bare QCM substrates show very small variations (~0.1°), confirming the homogeneity of these surfaces. On the contrary, gold and silver supports with silver dendrites and nanoparticles show larger standard deviations, indicating substantial local variations in the contact angle. This finding confirms the high morphological inhomogeneity of these surfaces, as also observed in the SEM images. Understanding the surface hydrophobicity is also crucial to properly control both the droplet volume and the contact time when performing drop-casting of aqueous solutions on the sensor surface.

Reflectance spectra of the bare gold QCM supports (black line), AgDNs@Au support (blue line), and gold QCM support electrodeposited with the second procedure (red line) were acquired and converted into absorption coefficients (α) using the Kubelka–Munk equation [36]. The resulting spectra are shown in Figure 3b. As observed, the spectra obtained for the bare gold QCM substrate and for the one modified with the second procedure (red line) are very similar, providing further confirmation that the electrodeposition did not proceed as expected, as also observed in the SEM images and wettability measurements. The blue line in Figure 3b, related to AgNDs@Au, shows a broad absorption band with three primary contributions centered at approximately 525, 585, and 685 nm. This behavior indicates the presence of multiple silver nanoparticle populations differing in shape and size, while the broad background underlying the three peaks was attributed to dendritic silver structures. These structures consist of trunks and branches of different sizes due to the random nature of their formation and growth mechanism for diffusion-limited aggregation (DLA) [47], and this architecture leads to multiple overlapping plasmonic absorption modes, creating a broad absorption feature that extends across the entire visible range. This interpretation is supported by related experimental findings in the literature [48].

Assuming that the three distinct peaks can be attributed to quasi-spherical silver nanoparticles (AgNPs), we realized a calibration curve (Figure S5) using previously published size–absorption data for AgNPs dispersed in water [48], and details of the computations are reported in the SI. Applying the corrected calibration fit to our data, we estimated the diameters of the three AgNPs populations on the surface of the AgNDs@Au support, which were found to be approximately 295 nm, 385 nm, and 535 nm. The sizes of the three AgNPs populations estimated from the reflectance spectrum are in good agreement with the dimensional analysis performed on the SEM images using the Image software, as shown in Figure S4, suggesting that reflectance spectroscopy flanked by contact angle measurements may represent a fast but reliable screening procedure for electrodeposition samples.

#### 3.1.3. SERS Characterization of Electrodeposited Plasmonic Nanostructures

SERS experiments on AgNPs electrodeposited on ITO slides were carried out using Rhodamine 6G (R6G) as a model analyte due to its well-known Raman features and its strong fluorescence [49]. Two silver-electrodeposited ITO substrates were tested: one with nanoflower (AgNFs) and the other with nanodendrites (AgNDs); the slides were exposed to R6G water solutions at different concentrations (from 10^−6^ to 10^−10^ M). All measurements were performed under resonant Raman conditions (laser excitation = 514 nm), resulting in Surface-Enhanced Resonant Raman Scattering (SERRS) effect. The resulting spectra are reported in Figure S6A,B in the Supplementary Materials.

The AgNFs substrate showed a good signal enhancement; however, it suffered from a significant fluorescence background at higher R6G concentrations, likely due to residual PEG 400 acting as a spacer and limiting the proximity between analyte molecules and the metallic surface. At lower concentrations, the signal-to-noise ratio improved, although fluorescence quenching remained partial (Figure S6A). In contrast, the AgNDs substrate delivered superior performance, enabling clear detection of characteristic R6G peaks even at 10^−10^ M (Figure S6B). This enhanced sensitivity is attributed to the absence of PEG and the larger effective surface area of the dendritic structures, which promotes better analyte adsorption and more effective fluorescence quenching. Strong and reproducible SERRS signals were obtained even at reduced laser powers, confirming the high efficiency and robustness of these nanostructures. Based on these results and considering both the analytical performance and the improved synthetic compatibility with QCM supports, we selected the AgNDs-based nanostructures for further studies involving ultrasensitive detection of FKBP12 via SERS, as described in the following sections.

### 3.2. Formation of Self-Assembled Monolayers Containing the Receptor for FKBP12 on QCM Supports

#### 3.2.1. QCM Monitoring of the Formation of Self-Assembled Monolayers Containing GPS-SH1

We studied the formation of SAMs of the GPS-SH1 receptor for FKBP12 on bare gold (Au) and bare silver (Ag) QCM supports as well as on gold QCM supports functionalized with electrodeposited silver nanostructures (AgNDs@Au). The results were comparatively analyzed to understand the behavior of the receptor GPS-SH1 on different metallic surfaces.

**Figure 4:**
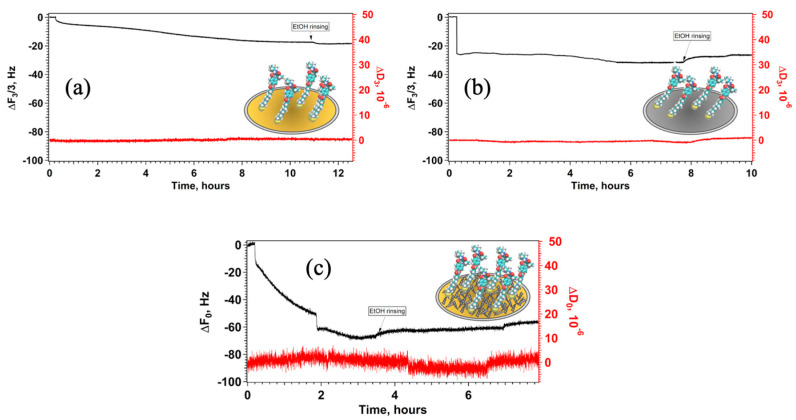
Variation in the normalized frequency and dissipation during the formation of SAMs composed by GPS-SH1 on bare gold (**a**), bare silver (**b**) and AgNDs@Au and (**c**) QCM supports.

Figure 4 shows the change in ΔF_n_/n and ΔD as a function of time after the addition of a 1 mM solution of GPS-SH1 in ethanol in the measuring chamber.

The decrease in ΔF_n_/n over time reaches a saturation point in all cases indicating the formation of a monolayer in the available surface; interestingly the kinetics vary depending on nature and morphology of the surface. Rinsing with ethanol removes any non-adsorbed molecules from the boundary layer close to the sensor surface, resulting in the formation of a complete self-assembled monolayer (SAM). For the flat sensors, the third harmonic (ΔF_n_/3) was evaluated, commonly used for bare QCM sensors because it provided a lower noise level compared to the fundamental frequency (F_0_); instead, for the nanostructured supports, we choose to use the fundamental frequency (F_0_). This choice was based on two main considerations: First, the F_0_ value from AgNDs@Au QCM supports showed a lower signal-to-noise (S/N) ratio than F_3_. Secondly, F_0_ enables the analysis of a thicker boundary layer, which is more appropriate for nanostructures such as silver dendrites formed on the sensor surface. Using F_3_ in this system would lead to an underestimation of the adsorbed mass, as molecules bound to the tips of the dendrites, extending farther from the surface, are not effectively detected at higher harmonic frequencies.

Analysis of the QCM results provided the structural parameters for the SAMs of GPS-SH1 on all investigated surfaces. The values reported in Table 1 show that, on flat QCM supports, GPS-SH1 adsorption is higher on silver compared to gold, and the resulting monolayer is more compact. 

Moreover, the tilt angle, defined as the angle between the molecule’s long axis and the surface normal, is greater for GPS-SH1 chemisorbed on gold (tilt angle_Au_ = 43°, tilt angle_Ag_ = 34°). This is ascribed to the different spacing of the metal surfaces, which results in a tighter packing of sulfur groups on gold. This observation is consistent with previous reports on SAMs formed by long-chain thiols [50].

On the AgNDs@Au supports, GPS-SH1 adsorption is more than three times higher than on flat sensors, demonstrating a promising result, as a larger amount of immobilized receptor may lead to greater immobilization of the FKBP12 protein, potentially improving both the sensitivity and the detection limit of the proposed sensing platform. This is consistent with the surface coverage results, suggesting that the branched structure of the dendrites, with their multiple orientation, allows for the binding of multiple GPS-SH1 molecules.

#### 3.2.2. Raman Identification of GPS-SH1 on SAM-Coated QCM Supports

Preliminary Raman characterization of the bulk GPS-SH1 receptor was performed on samples obtained from drop-casting GPS-SH1 dissolved in a small volume of ethanol onto a microscope slide followed by solvent evaporation. This process was repeated multiple times to accumulate enough material for analysis and residues of ethanol-related signals were removed by spectral subtraction.

**Figure 5:**
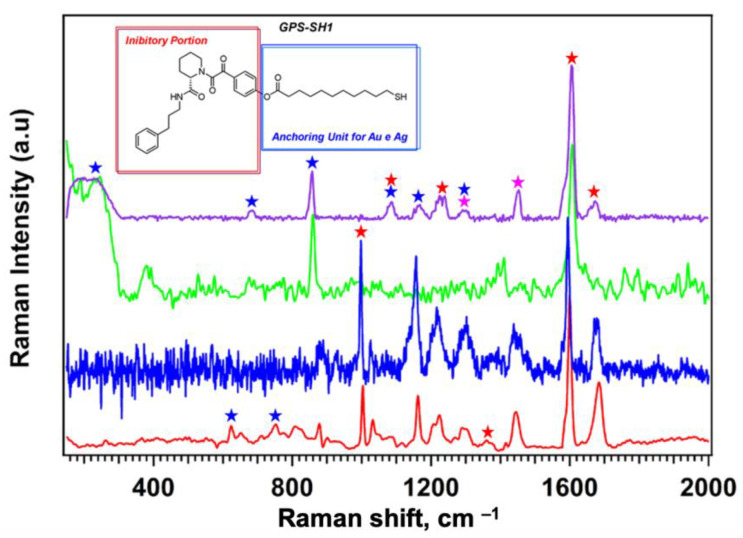
Raman spectra of the GPS-SH1 receptor acquired under different conditions. **Red**: FT-Raman spectrum of bulk GPS-SH1 (1064 nm laser, 200 mW, 1000 scans). **Blue**: Micro-Raman spectrum of bulk GPS-SH1 (785 nm laser, 750 μW, 20× objective, 15 s × 5 scans). **Green**: FT-Raman spectrum of GPS-SH1 SAM on Au/QCM with Ag nanostructures (1064 nm laser, 90 mW, 500 scans). **Purple**: SERS-enhanced micro-Raman spectrum of GPS-SH1 SAM (514 nm laser, 375 μW, 50× objective, 20 s × 3 scans).

In the spectra reported in Figure 5, the red trace corresponds to the FT-Raman spectrum of bulk GPS-SH1, measured using a laser with a 1064 nm excitation wavelength. The blue trace shows the micro-Raman spectrum of the same bulk sample, acquired using a 785 nm laser. The Raman spectra show the same features, suggesting that GPS-SH1 is also detectable by micro-Raman technique.

The Raman signal assignments for the GPS-SH1 molecule are reported in Table S2 The signal assignments are color-coded based on the region of the molecule from which they originate. Signals in blue correspond to the linear chain ending with the thiol group (–SH), which is responsible for anchoring the molecule to the plasmonic surface of the QCM support. Signals in red refer to the portion of the molecule involved in the recognition and coordination of the FKBP12 protein. Magenta highlights indicate signals common to both regions of the molecule. To facilitate interpretation, the structure of GPS-SH1 is shown in Figure 5, highlighting the two distinct regions discussed. Separate experiments on bulk 1-Dodecanethiol, in agreement with literature reports [51], allowed us to locate the positions of the main bands of the alkyl linker of the GSP-SH1 molecule. Examples of signals assigned to the linear chain include those related to the Ag–S and C–S bonds.

The signals corresponding to the portion of GPS-SH1 involved in the coordination of FKBP12 protein are found in the spectral region above 1000 cm^−1^. These include bands related to aromatic rings and amide groups, including the symmetric aromatic ring breathing at 1000 cm^−1^, C–N stretching in the piperidine ring and carbamoyl group at 1220 cm^−1^, and the amide II (N–H bend + C–N stretch) and aromatic ring C=C stretch at 1360 and 1580 cm^−1^.

The FT-Raman spectrum (1064 nm laser) of GPS-SH1 SAM built on AgNDs@Au (green trace) and the SERS-enhanced Raman spectrum of the same SAM (purple trace) are also reported in Figure 5. In this case, the total GPS-SH1 density determined by QCM was approximately 10^15^ molecules·cm^−2^ distributed between the dendritic structures, the nanospheres, and partly on the flat gold surface. The FT-Raman spectrum acquired with a 1064 nm excitation laser shows only a few Raman bands. In contrast, the micro-Raman spectrum, collected with a shorter wavelength laser and tightly focused spot, reveals a rich vibrational profile. This difference can be attributed to the limited SERS enhancement at 1064 nm, as this wavelength is far from the plasmon resonance of silver nanostructures. Additionally, the micro-Raman setup enables efficient laser focusing on SERS-active “hot-spots”, significantly enhancing the signal. The larger laser spot in FT-Raman, combined with the lower Raman scattering efficiency at longer wavelengths, results in a much weaker spectral response.

By comparing the spectra in Figure 5 with the assignments in Table S2, it is evident that the broad band around 240 cm^−1^, assigned to the Ag–S bond, appears only in the spectra of the GPS-SH1 SAM on the gold QCM substrate decorated with silver nanostructures. This low-frequency signal confirms the successful functionalization of the QCM surface with GPS-SH1. Another key difference between the bulk and SAM spectra concerns the stretching vibration of the C–S bond. This signal is clearly visible in the purple spectrum (SERS, ~685 cm^−1^), while it is very weak or absent in the others. This difference is due to the SERS effect, as the C–S bond is located close to the plasmonic surface and is therefore strongly enhanced. The position of this band also provides structural information. The C–S stretching typically appears at lower wavenumbers (~650 cm^−1^) for the gauche conformation and at higher values (~700 cm^−1^) for the trans conformation. In this case, the band is observed at an intermediate position (~685 cm^−1^), suggesting that the linear GPS-SH1 chain adopts a partially folded conformation. This indicates that the SAM formed by GPS-SH1 alone is not fully compact and that the receptor chain is not entirely extended.

The bands assigned to the GPS moiety are clearly visible in both the bulk GPS-SH1 spectra and in the SAM spectrum on the plasmonic surface. When comparing the bulk spectra (red or blue) with the SAM spectrum (purple), small shifts in the position of some bands are observed as commonly observed for SERS spectra [29]. In addition, variations in the relative intensity of some bands are observed, which may be related to SERS amplification effects and to conformational changes of the molecule upon surface binding. For example, the band at 1600 cm^−1^ is intense in both the bulk and SAM spectra, whereas the band at 1000 cm^−1^ disappears in the GPS-SH1 SAM spectra.

### 3.3. FKBP12 on QCM Functionalized with Dendritic Nanostructures

#### QCM Detection of FKBP12

We prepared mixed SAM of GPS-SH1 and an inert scaffold since in previous experiments by our group [23,24] identified 1-Dodecanethiol (C_12_-SH) and thiol-polyethylene glycol (PEG-SH) as promising anti-fouling scaffolds, with an optimal mixing ratio of the inert molecules to protein receptor (GPS-SH1) of 6:1. The addition of C_12_-SH and PEG-SH in specific quantities has a double role: it prevents interactions between GPS-SH1 molecules that could hinder protein binding and reduces the non-specific adsorption of interfering protein. Based on these findings, we functionalized two new AgNDs@Au QCM supports with mixed SAMs containing the GPS-SH1 receptor and either C_12_-SH or PEG-SH as anti-fouling components in a 1:6 molar ratio. The details of this functionalization process and the extensive characterization of the resulting platforms were previously reported in a preliminary work [26]. As discussed there, the presence of the nanoplasmonic structures enhances both sensitivity and the limit of the detection within the linear response range.

**Figure 6:**
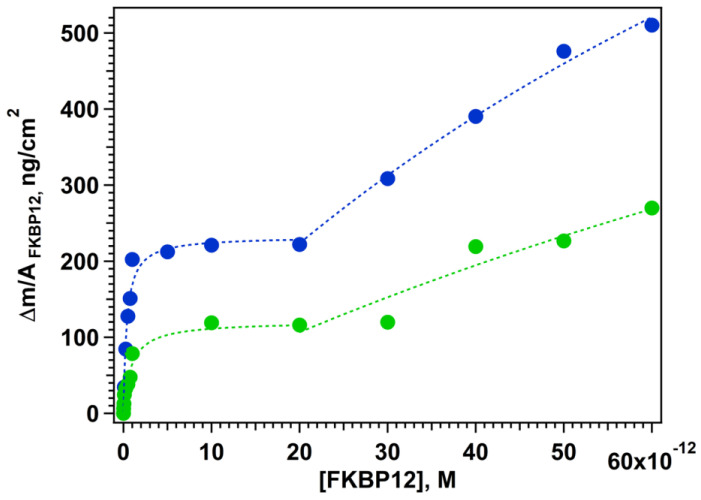
Double-step absorption of FKBP12 on AgNDs@Au QCM sensors with SAM of C12-SH/GPS-SH1 6:1 (in **blue**) and PEG-SH/GPS-SH1 6:1 (in **green**).

In the present work we explored a larger range of FKBP12 concentrations, and we observed two distinct adsorption steps for both SAMs reported in Figure 6.

The first step is rapid and corresponds to saturation of high-energy sites at low FKBP12 concentrations. The second step is slower and corresponds to the binding on the lower-energy areas. Similar differences in the kinetic of adsorption were observed also in the formation of SAMs of GPS-SH1/C12SH on flat and nanostructured surfaces, likely due to the same heterogeneity in nanoparticle morphology. In the range of FKBP12 concentrations explored, we assume that all available binding sites with different surface energies are fully saturated. We cannot exclude the possibility of a third phase, where residual adsorption occurs directly on the exposed gold surface at higher FKBP12 concentrations. We recall that SEM analysis evidenced that ca. 60% corresponds to fragmented flat surfaces scattered among the AgNPs. The two-step behavior likely reflects differences in surface energy between the nanostructured sensors. Flat QCM sensors present a uniform surface with consistent energy across the surface. In contrast, the AgNDs@Au sensors exhibit areas with varying surface energies: high-energy regions at the dendrite tips, and lower-energy areas on flatter regions containing more spherical AgNPs.

Both adsorption curves in Figure 6 were fitted with a modified multiple-site Langmuir equation that describes the adsorption of one species to two or more distinct types of adsorption sites. Provided that each binding site is not correlated to the other, the chemisorption is described as a sum of Langmuir expression. This approach was reported also by other authors for the adsorption of drugs on heterogeneous surfaces [52].

Best results were obtained fitting the data with the two-step Langmuir Equation (2) as shown in Figure 6.

(2) $${\left(∆m/A\right)}_{tot}=\frac{{\left(∆m/A\right)}_{1}^{max}[FKBP12]\;}{1+K_{1}[FKBP12]}+\;\frac{{\left(∆m/A\right)}_{2}^{max}[FKBP12]\;}{1+K_{2}[FKBP12]}$$

where (Δ*m*/*A*)_tot_ is the total measured surface density, $K_{1}$ = 1/*K*^1^*_d_* and $K_{2}$ = 1/*K*^2^*_d_* are the affinity constant for the two steps, and ${\left(∆m/A\right)}_{1}^{max}$ and ${\left(∆m/A\right)}_{2}^{max}$ are the surface density at each plateau. A summary of the fitting parameters is reported in Table 2.

The two dissociation constants obtained from the Langmuir fits (*K*^1^*_d_* and *K*^2^*_d_*) provide a clear molecular interpretation of this behavior. *K*^1^*_d_* is significantly lower than *K*^2^*_d_*, describing the initial, rapid binding step, in which high-energy surface sites (AgNDs) are saturated at low FKBP12 concentrations. *K*^2^*_d_*, by contrast, reflects a slower and lower-affinity interaction occurring on surface regions with lower energy (AgNPs). The results shown in Table 2 confirm that our previous findings [26] for the first adsorption step and the dissociation constants found previously for Au flat surface [26] are *K_d_* (GPS-SH1/C12-SH 1:6) = 811 pM and *K_d_* (GPS-SH1/PEG-SH 1:6) = 54 pM, suggesting that for the GPS-SH1/C12-SH 1:6 SAM, comparison with the present data suggest that in the case of GPS-SH1/C12-SH 1:6 SAM, two AgNP populations mainly contribute to surface adsorption in this FKBP concentration range, i.e., AgNDs and AgNs.

Moreover, analysis of low FKBP12 concentration regions confirmed that the AgNDs@Au QCM support functionalized with SAM of GPS-SH1/C12-SH 1:6 achieved a limit of detection (LOD) of 0.2 pM, demonstrating its outstanding potential for ultra-sensitive detection of FKBP12. These findings highlight the importance of surface design and receptor presentation in optimizing sensor performance.

### 3.4. SERS Detection of FKBP12

Given the body of the QCM results, we selected the AgNDs@Au QCM support functionalized with SAM of GPS-SH1/C12-SH 1:6 for in situ detection of FKBP12 with SERS experiments.

#### 3.4.1. SERS Characterization of Free and ELTEN378-Bound FKBP12

Preliminarily, we characterized the Raman spectrum of the FKBP12 protein that, to the best of our knowledge, has not been reported yet. The FKBP12 protein, whose primary sequence is reported in the Supplementary Materials, exhibits a well-defined secondary structure, primarily composed for a five-stranded antiparallel β-sheet and a short α-helix. The β-sheet follows a +3, +1, –3, –1 topology and includes the following strands: β1 (Val2–Ser8), β4 (Arg71–Ile76), β5 (Leu97–Leu106), β2 (Thr21–Leu30), and β3, which is split into two segments (Lys35–Ser38 and Phe46–Met49). The α-helix, spanning residues Ile56 to Val63, is amphiphilic and interacts with the hydrophobic face of the β-sheet, contributing to the formation of the protein’s hydrophobic core [2]. This arrangement stabilizes the overall fold and creates specific vibrational environments detectable by Raman spectroscopy.

**Figure 7:**
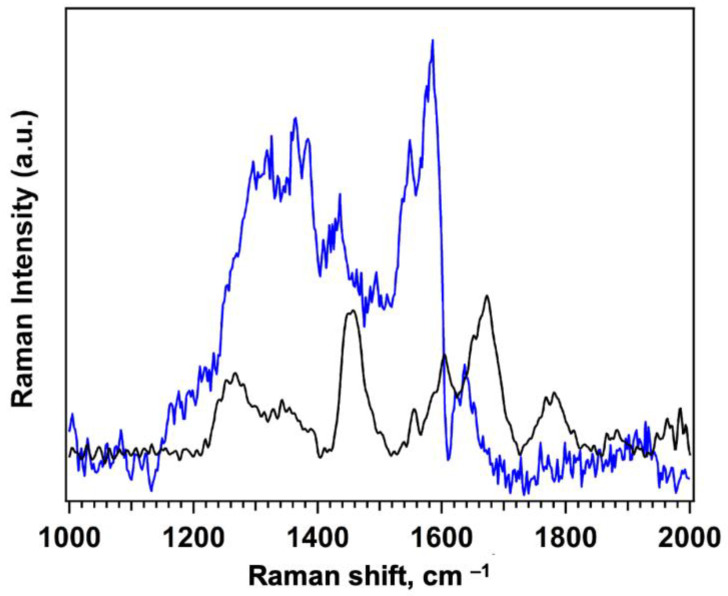
FT-Raman spectrum (black line; 1064 nm laser, 150 mW) and SERS spectrum (blue line; 514 nm laser, 750 μW, 50× objective, 20 s × 3 scans) of the bulk receptor-free FKBP12 protein.

The FT Raman spectrum of the protein was recorded on lyophilized FKBP12 sample prepared from the stock solution described in the Materials and Methods section (C = 2.4009 × 10^−5^ M in PBS: 0.5 M KH_2_PO_4_/K_2_HPO_4_ and 0.15 M NaCl, pH 7.4). Lyophilization was employed to remove all the water and concentrate the protein, allowing direct analysis of the solid phase. The buffer spectrum was also recorded and subtracted from the FKBP12 spectrum to eliminate background contributions. Additionally, SERS measurements were performed by drop-casting FKBP12 (C= 1 × 10^−6^ M) onto ITO substrates functionalized with silver nanodendrites (AgNDs). A comparison between the Raman and the SERS spectra of free bulk FKBP12 is shown in Figure 7. The spectra reveal the main Raman peaks in the fingerprint region commonly associated with protein vibrational modes. Notably, the SERS spectrum shows significantly enhanced intensity due to the plasmonic properties of the nanostructured AgNDs. However, in the absence of immobilization, the protein’s interaction with the SERS-active surface is limited, resulting in high spectral noise. This highlights the importance of immobilizing FKBP12 on the synthetic receptor, as implemented in the QCM setup, to enhance signal strength and reproducibility, as discussed in Section 3.4.2.

The Raman spectrum of FKBP12 reflects its molecular architecture, with characteristic vibrational bands arising from both backbone and side-chain motions. In particular, the amide I (1650–1670 cm^−1^) and amide III (1250–1280 cm^−1^) bands, which are sensitive to the protein’s secondary structure, are clearly observed and attributed to vibrations of the peptide backbone. In this region, distinct peaks due to the presence of aromatic residues such as phenylalanine, tyrosine, and tryptophan are very weak. Furthermore, the band at 1455 cm^−1^ was attributed to the symmetric stretching vibrations of carboxylate (COO^−^) groups, typically found in the side chains of aspartic acid and glutamic acid residues. A detailed assignment of the main Raman bands is provided in Table S3 (Supplementary Materials).

The SERS spectrum of the receptor-free FKBP12 deposited on AgNDs (blue line in Figure 7) exhibits, as expected, noticeable shifts compared to the FT Raman spectrum. The shifts can be attributed to the intrinsic nature of SERS that can selectively amplify specific vibrational modes, leading to apparent spectral shifts and intensity changes when compared to Raman spectra. Moreover, residual solvation water may remain adsorbed onto the protein despite partial evaporation, potentially preserving a more flexible or hydrated conformation. This difference in hydration and the conformational variations can affect the exposure and orientation of functional groups interacting with the AgND surface, thereby altering the vibrational profile and contributing to spectral shifts. Interestingly, in both spectra we observed a characteristic band located at 1430–1450 cm^−1^ assigned to the ASP/Glu residues.

We also tentatively investigated the interaction between the FKBP12 protein and its inhibitor ELTEN378 [53], which shares with GPS-SH1 the same active binding portion for protein interactions.

FKBP12 (C = 1 × 10^−6^ M) and ELTEN378 (C = 4 × 10^−9^ M) were co-deposited from solution onto AgNDs functionalized ITO substrates. The comparison between the SERS spectrum of free FKBP12 (blue) and that of FKBP12 complexed with ELTEN378 are shown in Figure S7. The comparison did not reveal significant spectral differences. This is likely due to the low concentration of the receptor in the mixture and the random orientation adopted by FKBP12, whether free or complexed, upon non-specific adsorption onto the plasmonic surface following drop-casting. These findings further support the need for a receptor-functionalized SAM to achieve stable and reproducible protein immobilization for SERS analysis.

#### 3.4.2. SERS Analysis of FKBP12 Protein Adsorbed on the QCM Functionalized with Nanodendrites

We preliminarily performed parallel SERS experiments on SAM (GPS-SH1/C_12_-SH 1:6 at gNDs@Au QCM without the addition of FKBP12. The characteristic SERS peaks of the mixed GPS-SH1/C_12_-SH 1:6 SAM) compared to the SERS peaks of GPS-SH1 SAM are reported in Table S4 in the Supplementary Materials. As observed from the band assignments, the spectra are similar but not perfectly overlapping. The differences can be attributed to the presence of dodecanethiol in the mixed SAM, whose Raman bands are reported in Figure S8 and assigned in Table S5 of the Supplementary Materials. These bands partially overlap with those of GPS-SH1, causing an apparent shift in some peaks. Additional differences between the two spectra arise from the different molecular orientations that the molecules can adopt in the two SAMs. Previous studies have shown that the mixed SAM is significantly more ordered than the pure GPS-SH1 SAM. This difference in ordering affects the conformation of the molecules and leads to shifts in specific bands, such as the C–S stretching mode, which shifts from 685 cm^−1^ in the more disordered SAM with predominantly gauche conformation to 715 cm^−1^ in the more ordered SAM with predominantly trans conformation.

A mixed self-assembled monolayer composed of GPS-SH1 and C12-SH (1:6 ratio) was prepared on a AgNDs@Au QCM support and a 60 pM FKBP12 solution was added to the measuring chamber. Protein binding to the immobilized receptor was monitored and followed by buffer rinsing. This approach reduces the degrees of freedom for protein orientation and promotes a more uniform and specific interaction between FKBP12 and the receptor bound to the surface. According to the results reported in Table 2, the FKBP12 density on the silver nanodendrites is expected to be ca. 220 ng/cm^2^, whereas 290 ng/cm^2^ of FKBP12 is immobilized on the AgNS population. A value of 220 ng/cm^2^ corresponds to approximately 1.1 × 10^13^ FKBP12 molecules/cm^2^. By knowing the laser spot size used in our micro-Raman spectroscopy setup (~2 µm), we can then estimate the average number of FKBP12 molecules being probed in a SERS experiment. We also underline that the surface density is correlated to the bulk concentration of the protein in the solution to be analyzed. As a result, with this approach we can precisely determine the amount of protein present on the surface that is effectively being probed by the SERS analysis.

**Figure 8:**
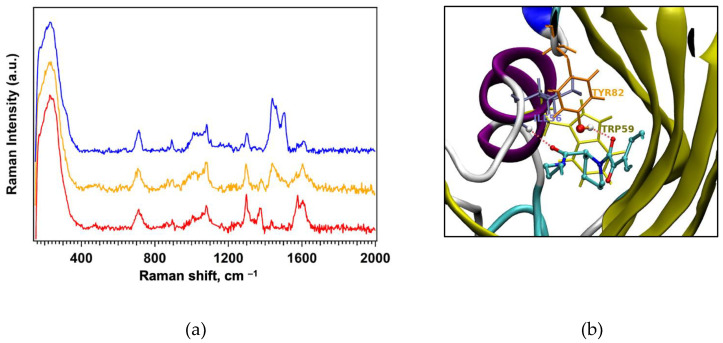
(**a**) SERS spectra of GPS-SH1/C12-SH 1:6 SAM after exposure to FKBP12 followed by ethanol immersion for 0 h (blue), 40 h (orange), and 14 days (red). (**b**) GPS receptor functionality binding in the FKBP12 pocket. All spectra were acquired under the same experimental conditions: 514 nm laser, 375 μW, 50× objective, 20 s × 3 scans.

The SERS spectrum of the captured protein shown in Figure 8a (blue trace) exhibits the characteristic peaks of GPS-SH1 and distinct peaks at 1438 and 1504 cm−^1^ assigned to the protein. Interestingly, the intensity of some of the peaks assigned to the Amide II (N–H bend + C–N stretch), aromatic ring C=C stretches of GPS-SH1 undergo a significant decrease in intensity upon binding to the protein. Actually, the chemical functionalities involved in the binding to the proteins are described in Figure 8b: the FKBP12-binding group of GPS-SH1 is represented in CPK with standard colors for the atoms. TRP59, stacked to the pipecolic ring, is reported in yellow, whereas ILE56 and TYP82 are in ice blue and orange colors, respectively. The two H-bonds involving the NH backbone moiety of ILE56 and the oxydryl of TYR82 with the carbonyl groups of the amide moieties of N-ELTEN378 are shown with a red dashed line. These two persistent donated H-bonds by ILE56 and TYR82 [54] subtract electron density from the amide conjugated bond, thus reducing the Raman intensity of the associated modes when the GPS moiety is interacting with FKBP12. 

These findings suggest that while the 1430 cm^−1^ band associated with the ASP/Glu residues of FKBP12 may serve as marker for the protein, the suppression of the bands associated to the GPS group confirms the presence of the bound protein and the selectivity of the detection since common protein interferents in biological fluids do not bind to GPS-SH1 [23,24].

The same FBP12-bound sensor was further immersed in ethanol, and SERS spectra were recorded at increasing immersion times. This process yielded the orange and red spectra also reported in Figure 8, corresponding to intermediate and longer ethanol exposure times, respectively. As reported in our earlier studies [23,24] ethanol immersion leads to protein denaturation and to its gradual removal from the sensor surface.

All spectra reported in Figure 8 were normalized with respect to the vibrational band at 230 cm^−1^, which is associated with the Ag–S interaction. This band exhibits very similar intensity across all acquired spectra, thus providing a reliable internal standard for normalization. Moreover, SERS measurements collected at multiple randomly selected points across the sensor surface yielded highly reproducible spectra, as illustrated in Figure S9 in the Supplementary Materials. This consistency further confirms the homogeneity of the sensor surface and the robustness of the detection method.

**Figure 9:**
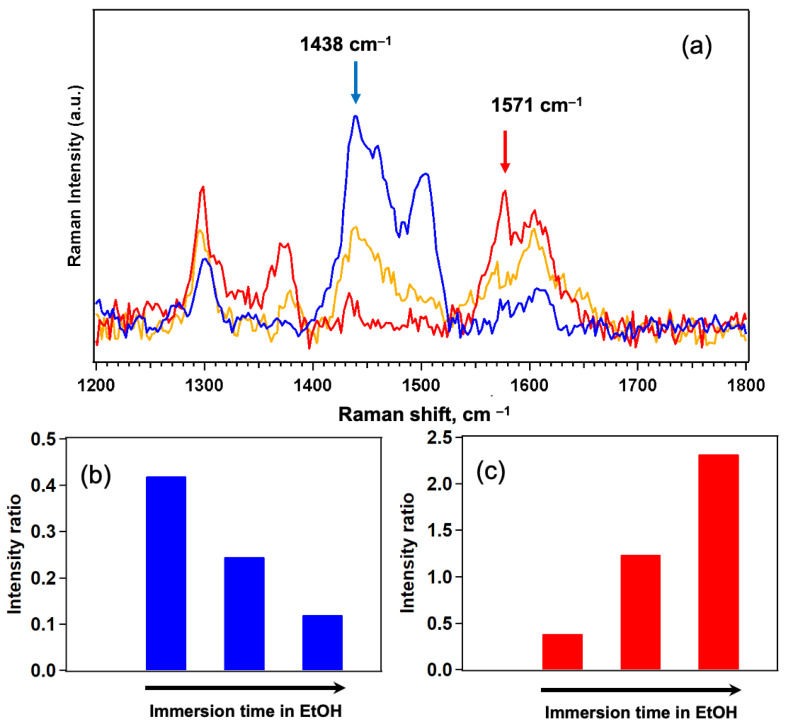
(**a**) Zoomed SERS spectra reported in Figure 8, arrows indicate FKBP12 (1438 cm^−1^) and GPS-SH1 (1571 cm^−1^) contributions. (**b**) Intensity ratios 1_438_/I_230_ and (**c**) I_1571_/I_1438_ during ethanol rinsing.

By comparing the evolution of the SERS spectra as a function of ethanol immersion time—and thus of the amount of protein released—we observe notable differences in the intensity of specific vibrational bands in the 1200–1800 cm^−1^ region. To facilitate the visualization of these spectral changes, a magnified view of the SERS spectra in the region of interest is provided in Figure 9. 

Specifically, as the ethanol immersion time increases, we observe a decrease in the intensity of the protein bands at 1438 cm^−1^ and 1504 cm^−1^, along with an increase in the intensity of bands at 1370 cm^−1^ and 1571 cm^−1^ of the GPS unit A quantitative analysis was carried out by focusing on the peaks at 1438 cm^−1^ and 1571 cm^−1^, but similar results were obtained also for the other two peaks.

The histograms shown in Figure 9b,c summarize these results: the blue bars represent the average intensity ratio of the 1438 cm^−1^ peak (normalized to the 230 cm^−1^ Ag–S band), which is attributed to FKBP12-related vibrations, likely arising from Asp and Glu residues (see Table S3). As shown in the figure, the intensity of the band decreases as the protein is released from the sensor upon ethanol denaturation. The red bars indicate the average ratio between the receptor-associated band at 1571 cm^−1^ (assigned to Amide II and aromatic C=C stretching modes) and the FKBP12 signal at 1438 cm^−1^, and this ratio monitor changes in the receptor as a function of protein coverage. As illustrated in Figure 8b, the amide and aromatic groups of the receptor are involved in the interaction with the FKBP12 protein. Upon complexation, we expect a reduction in the vibrational degrees of freedom of these functional groups due to binding constraints, which results in a decrease in their corresponding Raman signal intensity. This phenomenon is reflected in the red histogram: as the protein is progressively released during ethanol immersion, the vibrational freedom of the amine and aromatic groups is restored, leading to a gradual increase in the Raman intensity of the associated peaks. All reported ratios are calculated as the average over multiple spectra acquired at randomly selected positions on the sensor surface, further confirming the high reproducibility and spatial homogeneity of the SERS response.

Overall, these findings demonstrate that SERS detection of FKBP12 is feasible using a GPS-SH1/C_12_-SH-functionalized plasmonic sensor. Although the direct identification of all protein bands remains challenging due to spectral overlap, the receptor’s vibrational changes upon protein binding provide a clear ratiometric parameter that flanked by quantifiable protein signatures at 1438 cm^−1^ represent a clear concentration dependent fingerprint of the FKBP12 protein. This approach offers a promising route for label-free protein sensing, and future work will focus on enhancing sensitivity and enabling quantification through spectral calibration curves.

## 4. Conclusions

In this work, we have successfully developed and characterized a novel dual-detection platform that synergistically combines quartz crystal microbalance (QCM) and surface-enhanced Raman spectroscopy (SERS) on a single sensor chip for comprehensive FKBP12 analysis. The platform’s key innovation lies in its ability to simultaneously quantify adsorbed protein mass while providing molecular-level structural information—a capability not achievable through conventional single-technique approaches.

The foundation of this system relies on our optimized silver nanodendritic structures, which were electrodeposited directly onto QCM substrates. Through systematic evaluation using Rhodamine 6G as a SERS probe, we identified the specific nanodendritic morphology that maximized both Raman enhancement and QCM stability. These nanostructures were subsequently functionalized with our patented thiolated receptor molecule GPS-SH1 mixed with an anti-fouling molecular scaffold, which forms a stable self-assembled monolayer while presenting selective FKBP12 binding groups toward the aqueous phase.

QCM measurements revealed distinctive two-stage Langmuir adsorption kinetics upon FKBP12 binding, enabling sensitive detection down to 0.2 pM. The simultaneous SERS measurements on the identical sensor surface provided complementary molecular information: characteristic FKBP12 vibrational bands were clearly resolved at concentrations as low as 20 pM. The specificity of these assignments was confirmed through ethanol denaturation controls, which caused complete disappearance of protein-related peaks while restoring the receptor’s original spectral signature.

Most significantly, we established a quantitative relationship between QCM-derived surface coverage and SERS response by developing a ratiometric parameter (I_1571_/I_1438_) that correlates linearly with adsorbed FKBP12 mass. This direct correlation between solution concentration, interfacial mass, and spectral response represents a major advance over traditional approaches that require separate measurements on different platforms.

The successful integration of QCM and SERS on a single chip, combined with our specialized receptor chemistry, creates a powerful new tool for studying protein–ligand interactions. Furthermore, the modular design principles demonstrated here—from tunable nanodendrite fabrication to adaptable receptor chemistry—suggest broad applicability to other clinically relevant protein targets.

## Figures and Tables

**Table 1:** Structural parameters of SAMs of GPS-SH1 on different QCM supports.

QCM Support	Δ*m*/*A*^a^ (ng/cm^2^)	Saturation Area (Å^2^/Molecules)	Thickness (nm)	ΔD (10^−6^)
*Au Flat*	323	31	19	1
*Ag Flat*	463	21	17	2
*AgNDs@Au*	1037	10	-	4

**Table 2:** FKBP12 detection parameters for different SAM compositions on QCM supports.

SAM Composition	*Δm/A* _experimental_ (ng/cm^2^)		*Δm/A* _theoretical_ (ng/cm^2^)		*K*^1^*_d_* (nM)	*K*^2^*_d_* (nM)
	*First step*	*Second step*	*First step*	*Second step*	*First step*	*Second step*
*GPS-SH1/C_12_-SH 1:6*	222.0	510.3	228.2	520.8	0.4 ± 0.1	119.1 ± 27.4
*GPS-SH1/PEG-SH 1:6*	116.1	269.9	115.8	268.6	0.9 ± 0.3	190.7 ± 253.0

## Data Availability

All data supporting the findings of this study are provided within the paper and its Supplementary Materials. All additional information will be made available upon reasonable request to the authors.

## Supplementary Materials

The following supporting information can be downloaded at: https://www.mdpi.com/article/10.3390/nano15161230/s1, Figure S1: SEM images at different magnification of silver nanoflowers (AgNFs) electrodeposited on ITO-covered glass. Figure S2: SEM images of silver nanostructures deposited changing the deposition times. Figure S3: SEM images of silver- and gold-coated QCM substrates. Figure S4: Size distribution of quasi-spherical AgNPs on gold-coated QCM support deposited with the first procedure described in the main text. Figure S5: Calibration curve showing the relationship between the diameter of spherical silver nanoparticles and the wavelength of their LSPR absorption maximum. Table S1: Corrected LSPR peak wavelengths and corresponding estimated diameters of AgNPs on gold-coated QCM supports. Figure S6: SERS spectra of R6G at different concentrations on the AgNF (A) and AgNDs (B). Table S2: SERS signal assignment of bulk and SAM GPSSH1 on Au QCM with silver nanostructures. Table S3: Peak assignments for Raman and SERS spectra of FKBP12 protein. Figure S7: SERS spectrum of the FKBP12 alone (blue) and in complex with ELTEN378 (red) on AgNDs-functionalized ITO supports. Table S4: SERS peak assignments for the mixed SAM composed of GPS-SH1 and C12SH in a 1:6 ratio, compared to the GPS-SH1 SAM alone. Figure S8: Raman spectra of 1-dodecanethiol. Table S5: Raman signal assignment of solid 1-dodecanethiol. Figure S9: SERS spectra of FKBP12 adsorbed on the SAM of GPS-SH1/C_12_-SH 1:6 ratio measured in several surface areas of the QCM support.
