# Supplementary material for: Combining QCM and SERS on a Nanophotonic Chip: A Dual-Functional Sensor for Biomolecular Interaction Analysis and Protein Fingerprinting
Source: Nanomaterials (Basel). 2025 Aug 12;15(16):1230. doi: 10.3390/nano15161230 (PMC12388188; doi:10.3390/nano15161230)
Supplement: Supplementary file 1 [file nanomaterials-15-01230-s001.zip › nanomaterials-3788063-supplementary-conversion.pdf]

Supporting Information

for

Combining QCM and SERS on a Nanophotonic Chip: A Dual-Functional Sensor for Biomolecular Interaction Analysis and Protein Fingerprinting

Cosimo Bartolini [1,2], Martina Tozzetti [1,2], Cristina Gellini [1], Marilena Ricci [1], Stefano Menichetti [1], Piero Procacci [1] and Gabriella Caminati [1,2,*]

[1] Department of Chemistry "Ugo Schiff", University of Florence, Via della Lastruccia 3-13, 50019 Sesto Fiorentino (FI), Italy
[2] Center for Colloid and Surface Science (CSGI), University of Florence, Via della Lastruccia 3-13, 50019 Sesto Fiorentino (FI), Italy
* Correspondence: gabriella.caminati@unifi.it

Silver nanoflowers (AgNFs) electrodeposited on ITO-coated glass using a three-electrode configuration. aA constant potential of −0.9 V was applied for 100 seconds in an electrolyte solution containing 0.5 mM $AgNO_3$ and 5 mg/mL PEG_{400}.

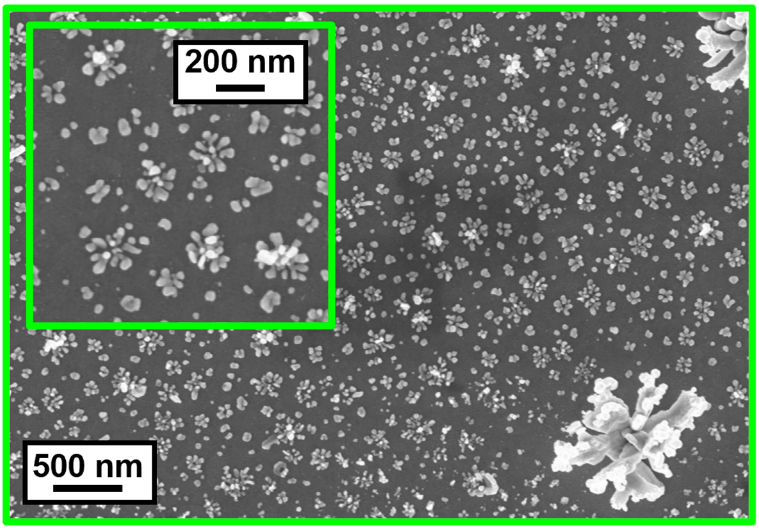

Figure S1. SEM images at different magnification of silver nanoflowers (AgNFs) electrodeposited on ITO-covered glass.

SEM images of two of ITO-coated glass samples electrodeposited changing the electrodeposition time using a low constant potential of -0.06 V in and an electrolyte solution composed of 5 mM $AgNO_3$ and 1 mM KNO_3. The SEM images highlighted in orange correspond to the sample deposited for a shorter time (120 s), while those highlighted in red refer to the sample deposited for a longer time (600 s).

The images reveal notable differences between the two. The 120 s sample shows no dendritic structures, but rather asymmetric nanoparticles with sizes between 200 and 400 nm. These nanoparticles serve as nucleation sites for the dendritic structures observed in the 600 s sample. The dendritic structures reach micrometer-scale dimensions, with branch cross-sections as small as ~30 nm. This morphological evolution depending on electrodeposition time is consistent with a Diffusion-Limited Aggregation (DLA) growth mechanism.

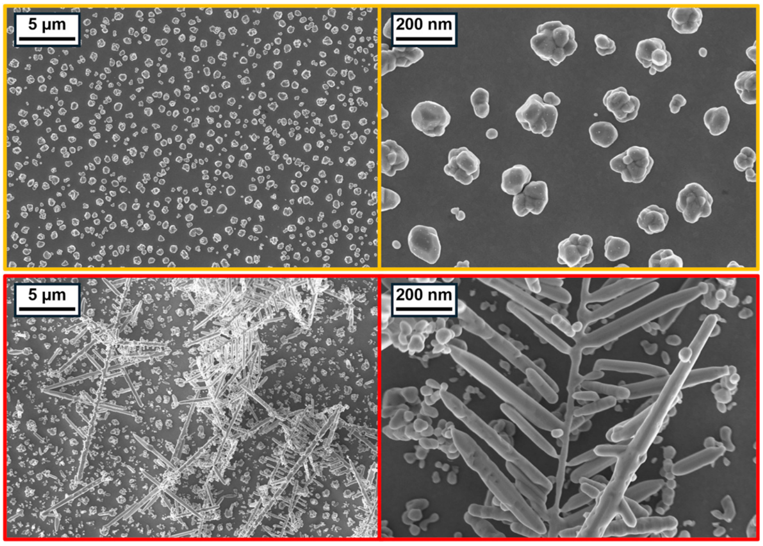

Figure S2. SEM images of silver nanostructures deposited changing the deposition times. The upper images (highlighted in orange) correspond to samples deposited for shorter times (120), showing only anisotropic nanoparticles. The lower images (highlighted in red) correspond to longer deposition times, resulting in well-developed dendritic structures.

Size distribution of quasi-spherical silver nanoparticles deposited on a gold-coated QCM substrate prepared following the first deposition procedure described in the main text. The size distribution was analyzed using a representative SEM image that corresponds to a surface region containing exclusively silver nanoparticles, with dimensions of 25 μm × 25 μm. The analysis yielded a particle size distribution that is reported in the histogram of figure S4.

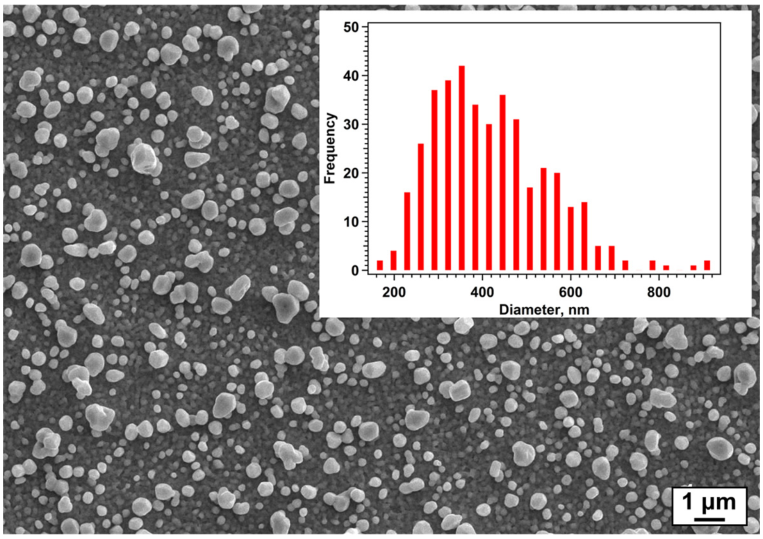

Figure S3. Size distribution of quasi-spherical AgNPs on gold-coated QCM support deposited with the first procedure described in the main text.

SEM images of silver- and gold-coated QCM substrates electrodeposited using the second procedure described in the main text. As observed, only a small number of AgNPs are visible, randomly and unevenly distributed across the surface. No well-defined nanostructures or flower-like morphologies are observed, indicating that this protocol is unfruitful for forming AgNFs on metallic QCM substrates.

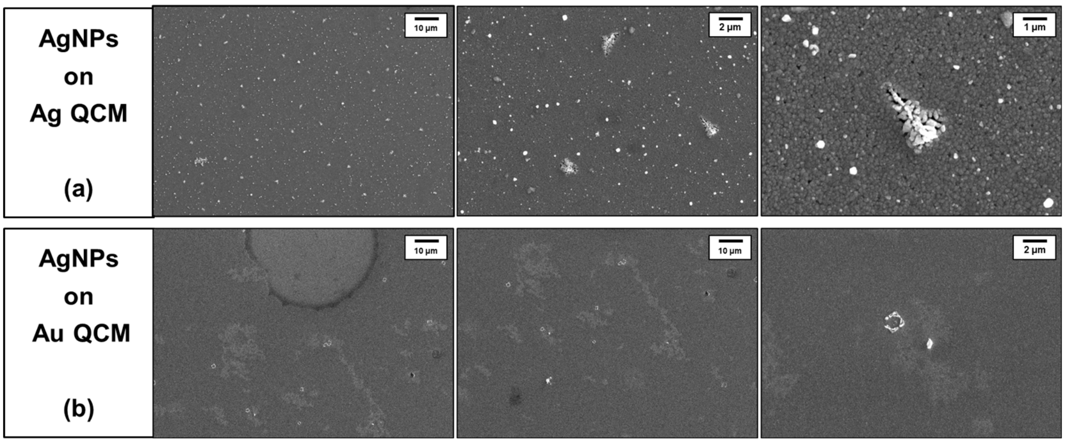

Figure S4. SEM images of silver- and gold-coated QCM substrates after applying the second electrodeposition procedure.

Calibration curve obtained by plotting the diameter of spherical silver nanoparticles as a function of the wavelength corresponding to their localized surface plasmon resonance (LSPR) absorption maximum. The data used to realise this calibration curve were extracted from literature [1] where UV-

Vis measurements were carried out on aqueous dispersions of monodisperse silver nanoparticles of well-known size.

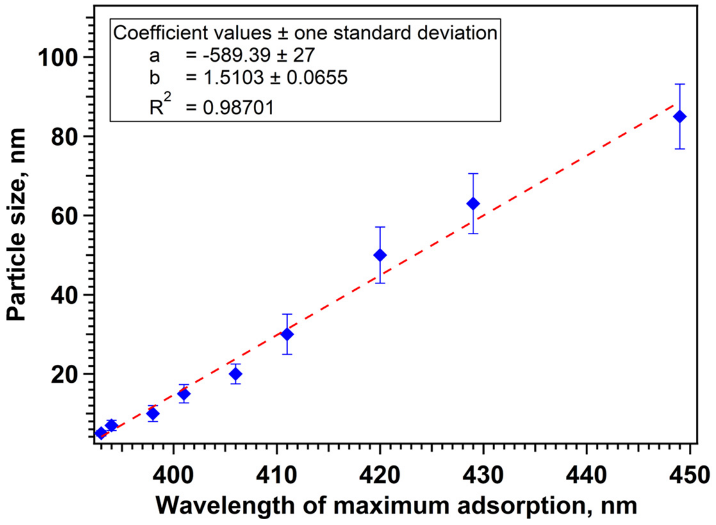

Figure S5. Calibration curve showing the relationship between the diameter of spherical silver nanoparticles and the wavelength of their LSPR absorption maximum, based on literature data [1].

Table S1. Corrected LSPR peak wavelengths and corresponding estimated diameters of AgNPs on gold-coated QCM supports treated with the first deposition procedure described in the main text.

AgNPs population	Experimental wavelength (nm)	Shifted wavelength (nm)	Nanoparticle estimated diameter (nm)
AgNP1	525	585	295
AgNP2	585	645	385
AgNP3	685	745	535

The first column reports the wavelengths of the LSPR maxima measured in air. To account for the difference in refractive index between air and water, a shift of +60 nm was applied to each value, as shown in the second column. The third column lists the estimated diameters of the corresponding silver nanoparticles, calculated using the calibration curve reported in Figure S5

Raman spectra of Rhodamine 6G (R6G) acquired at different concentrations using silver nanoflowers (**a**) and silver dendrites (**b**) as plasmonic substrates for surface-enhanced Raman scattering (SERS). The spectra were recorded in identical experimental conditions to compare the enhancement efficiency of the two nanostructured surfaces.

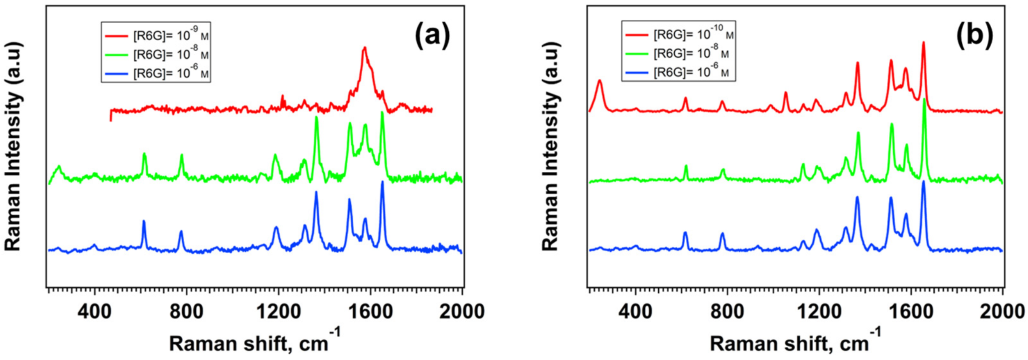

Figure S6. SERS spectra of R6G at different concentrations on the AgNFs (**a**) and AgNDs (**b**). Each spectrum was acquired under specific experimental conditions. AgNFs**:** 514 nm laser, 150 µW, 50x objective, 20 s × 3 scans (10^{-6} M); 514 nm laser, 150 µW, 50x objective, 20 s × 3 scans (10^{-8} M); 514 nm laser, 150 µW, 50x objective, 10 s × 10 scans (10^{-9} M). AgNDs: 514 nm laser, 150 µW, 50x objective, 10 s × 1 scan (10^{-6} M); 514 nm laser, 150 µW, 50x objective, 10 s × 1 scan (10^{-8} M); 514 nm laser, 150 µW, 50x objective, 10 s × 10 scans (10^{-10} M).

Table S2. SERS signal assignment of bulk and SAM GPSSH1 on Au QCM with silver nanostructures.

Raman shift (cm^{-1})				Assignment
FT Raman GPSSH1 bulk (laser 1064 nm)	Micro-Raman of bulk GPS-SH1 (laser 785 nm)	FT-Raman GPS-SH1 SAM on Au/QCM with Ag nanostructures (laser 1064 nm)	SERS-enhanced micro-Raman of GPS-SH1 SAM on Au/QCM with Ag (laser 514 nm)	
		245	240	Ag-S
623				C-C aliphatic chain
650			685	C-S stretching gauche
753				C-S stretching trans
810		860	857	CH_2 rocking/ C-O-C
1003	998			Symmetric aromatic ring breathing
1030	1026		1085	C-C trans stretching Aromatic ring
1160	1165		1166	C-C trans stretching
1220	1220		1230	C–N stretching in the piperidine ring and carbamoyl group
1300	1300		1300	CH_2 wagging, C-C gauche stretching
1360				Amide III (C–N stretch)
1445	1445		1453	CH_2 bending (alkyl chain, piperidine ring
1585 (shoulder)	1580 (shoulder)	1585 (shoulder)	1575 (shoulder)	Amide II (N–H bend + C–N stretch), aromatic ring C=C stretches of the piperidine ring
1600	1595	1607	1605	Aromatic C=C stretching (phenyl rings)
1684	1678		1674	Amide (C=O stretch) Carbamoyl (O=C–N)

Sequence of FKBP12

MGVQVETISPGDGRTFPKRGQTCVVHYTGMLEDGKKFDSSRDRNKPFKFMLGKQEVIRGWEEGVA QMSVGQRAKLTISPDYAYGATGHPGIIPPHATLVFDVELLKLE

Table S3. Peak assignments for Raman and SERS spectra of FKBP12 protein

Raman shift (cm^{-1})		Assignment
**FT RAMAN FKBP12 bulk (laser 1064 nm)**	**SERS FKBP12 on AgNDs (laser 514 nm)**	
1266	1313	Amide III (C-N stretch, N-H bend)
1341	1365	Tryptophan
1457	1429	ASP/Glu (COO^- stretch), CH_2 bend
1557	1549	Amide II (N–H bend + C–N stretch) + Trp W1 (indole ring stretch
1605	1585	Amide I (C=O stretch; α-helix/β-sheet N-H bend, C-N stretch)
1670	1637	Amide I (β turn, Random structure)

Comparison between the SERS spectrum of free FKBP12 and FKBP12 complexed with ELTEN378. Both measurements were performed on AgNDs fabricated on ITO substrates.

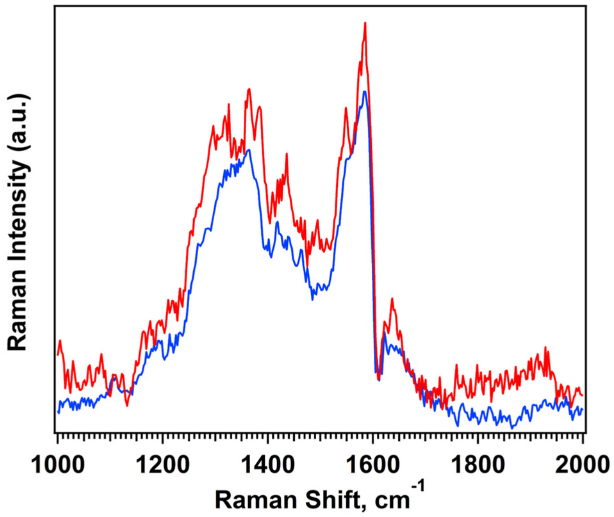

Figure S7. SERS spectrum of the FKBP12 alone (blue line; 514 nm laser, 750 µW, 50x objective, 20 s x 3 scans) and in complex with ELTEN378 (red line; 514 nm laser, 750 µW, 50x objective, 20 s x 3 scans) on AgNDs-functionalized ITO supports.

Table S4. SERS peak assignments for the mixed SAM composed of GPS-SH1 and C12SH in a 1:6 ratio, compared to the GPS-SH1 SAM alone. Both SAMs were formed on AgNDs@Au QCM support.

**Raman Shift (cm^{-1})**		**Assignment**
**Mixed SAM GPS-SH1/C_{12}SH 1:6**	**SAM GPS-SH1**	
240	***240***	**Ag-S**
	685	**C-S stretch gauche**
715		**C-S stretch trans**
870	***857***	**CH2 rock, C-O-C**
895		
1085	***1085***	**C-C stretching trans Aromatic ring**
1165	***1166***	**C-C trans streatching**
	1230	**C–N stretching in the piperidine ring and carbamoyl group**
1300	***1300***	**CH2 wagging, C-C gauche stretching**
1330		**CH_2 wagging**
1370		**amide III (C–N stretching)**
1435		**CH_2 bending (alkyl chain, piperidine ring)**
1455	***1453***	
1575	***1580 (shoulder)***	**Amide II (N–H bend + C–N stretch), aromatic ring C=C stretches**
1605	***1605***	**Aromatic C=C stretching (phenyl rings)**
1650 (shoulder)	***1674***	**Amide (C=O stretch)Carbamoyl (O=C–N)**

Raman spectra of bulk 1-dodecanthiol acquired using micro-Raman equipped with argon laser (emission λ=514.5 nm). Signal assignments of 1-dodecanthiol are reported in table S5.

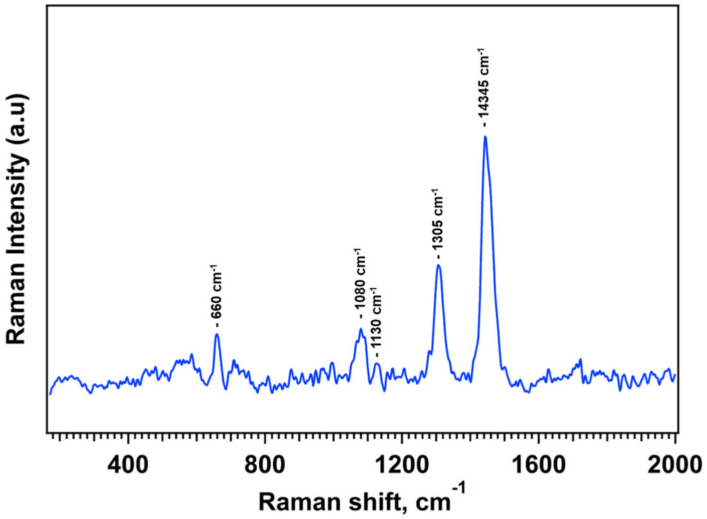

Figure S8. Raman spectra of 1-dodecanethiol (laser 514 nm, 750 μW, 20x objective, 20 s x 3 scans).

Table S5. Raman signal assignment of solid 1-dodecanethiol

Raman shift (cm^{-1}) solid 1-dodecanethiol from ref.	Assignment	Raman shift (cm^{-1}) solid 1-dodecanethiol measured	Assignment
654	ν(C–S)$_G$ stretching gauche	660	ν(C–S) stretching
1084	ν(C–C)$_T$ stretching trans	1080	ν(C–C) stretching
1130	ν(C–C)$_T$ stretching trans	1130	ν(C–C) stretching
1308	$\omega(CH_2)$ wagging	1305	$\omega(CH_2)$ wagging
1464	$\delta(CH_2)$ bending	1445	$\delta(CH_2)$ bending

Representative SERS spectra of FKBP12 adsorbed on a mixed self-assembled monolayer (GPS-SH1/C12SH, 1:6 ratio) formed on the AgNDs@Au QCM sensor surface. The spectra were acquired from randomly selected areas across the sensor surface, demonstrating the spatial reproducibility of the SERS signal.

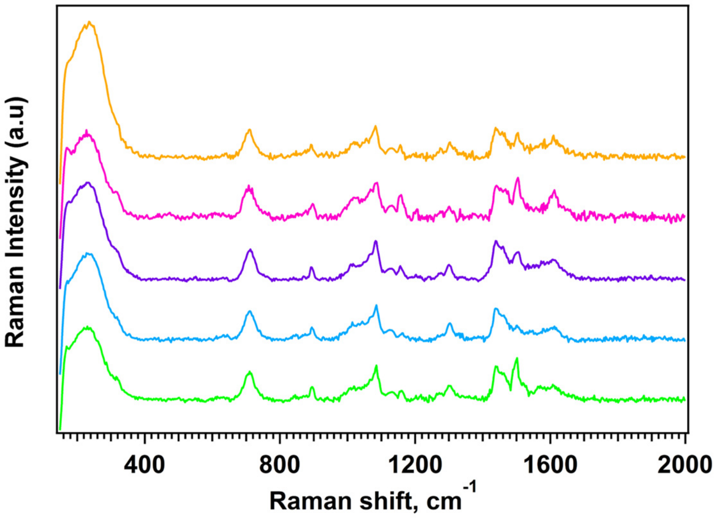

Figure S9. SERS spectra of FKBP12 adsorbed on the SAM of GPS-SH1/C_{12}-SH 1:6 ratio measured in several surface areas of the QCM support. All spectra were acquired under the same experimental conditions: 514 nm laser, 375 μW, 50x objective, 20 s x 3 scans.
